# Gender-specific association between uric acid level and chronic kidney disease in the elderly health checkup population in China

**DOI:** 10.1080/0886022X.2019.1591994

**Published:** 2019-04-11

**Authors:** Yanlang Yang, Wei Zhou, YuWei Wang, Ru Zhou

**Affiliations:** a Department of Nephrology, Affiliated Yijishan Hospital, Wannan Medical College, Wuhu, China;; b NanRui Community Health Service Centers, Wuhu, China

**Keywords:** Hyperuricemia, chronic kidney disease, gender, elderly

## Abstract

**Objective**: To evaluate the association between serum uric acid (SUA) levels with CKD in elderly health checkup population and explore the gender difference.

**Methods:** A total of 4242 subjects were included in the cross-sectional study. All of the subjects participated in the annual checkup between June 2016 and June 2017. Chronic kidney disease (CKD) was defined by estimated glomerular filtration rate (eGFR) <60 ml/min per 1.73 m^2^. We examined the association between SUA levels and CKD. Multivariate binary logistic regression analysis was used to estimate odds ratios (ORs) and 95% confidence intervals (95%CIs) by comparing association between the SUA level and CKD. The models were adjusted for age, gender, body mass index (BMI), hypertension, diabetes, triglyceride and high-density lipoprotein cholesterol (HDL-C).

**Result:** The prevalence of hyperuricemia was 22.2%, and it was significantly higher in male than in female (25.2% vs. 17%, *p* < .001). The prevalence of hyperuricemia increased with age, especially in the female. The prevalence of CKD was 27.8% in male and 20.2% in female (*p* < .001). Compared with the SUA first quartile, the multivariate-adjusted odds for CKD of fourth quartiles were 6.05 (95%CI: 4.32–8.49) in male and 8.21(95%CI: 5.37–12.54) in female.

**Conclusion:** Our finding showed gender-specific differences in the association between high SUA and an increased risk of CKD in the elderly population. The association of SUA and CKD was independent of other potential confounding factors including age, glucose, hypertension, HDL, TG and BMI.

## Introduction

Serum uric acid (SUA) is the end product of urine metabolism. Animal studies supported the role of uric acid as a mediator of risk for both hypertension and progressive decline in renal function [1]. Emerging evidence suggests hyperuricemia was associated with increasing risk of the incidence and progression of Chronic kidney disease (CKD) [2]. Meanwhile, hyperuricemia was an independent predictor of CKD in diabetes [3]. Possible mechanisms include: (1) the elevated SUA increases oxidative stress which decrease endothelial nitric oxide (NO) resulting in the induction of endothelial disfunction and podocyte injury [4]; (2) the elevated SUA activates the renin-angiotensin system [5], enhances the expression of adiponectin [6] and monocyte chemoattractant protein-1(MCP-1) [7] resulting in the inflammatory injury of renal proximal tubule; (3) the elevated SUA prompts vascular smooth muscle cell (VSMC) proliferation and vascular dysfunction of afferent arterioles resulting in renal hypoxia [8]. There were mixed results with some studies regarding the association of SUA with CKD in different gender in previous studies [5,9–11]. The elderly population had a higher level of SUA and a high risk of developing CKD [12]. However, as we know, there is little information about the association between hyperuricemia and CKD in the elderly population. In the present study, we sought to investigate the association of SUA and CKD in an elderly health checkup population in China. We also evaluate the association between SUA and CKD in different gender, respectively.

## Subjects and methods

### Subjects

This retrospective observational study used community health screening data in the NanRui Community Health Service Centers. This study protocol was approved by the research and ethics committee of the Yijishan Hospital of Wannan Medical College (Approval number: WY-IRB-2017-22).

All subjects were community-dwelling elderly from NanRui Community in Wuhu City, located in the eastern area of China. Totally, 4242 subjects were included in the present study, with 2700 males and 1542 females, respectively. The time span of the database was from June 2016 to June 2017. The average age of subjects was 67.11 ± 6.24 years (from 60 to 92 years). All subjects were categorized as five subgroups by their age (60–65 years, 66–70 years, 71–75 years, 76–80 years, and >80 years).

### Data collection

Before sampling collection, the subjects were required to have a low-fat diet without alcohol for dinner. After an overnight fast (at least 12 h), the venous blood samples were collected. Each subject was interviewed and completed a questionnaire including information on gender, date of birth and medical history. The height, body weight and blood pressure were manually measured by trained nurses.

Resting blood pressure was measured using a sphygmomanometer. Two blood pressure recordings were obtained from the right arm of subjects in a sitting position after 30 min of rest. The average of two measurements was performed. Height was measured in meters (without shoes), and weight was measured in kilograms (removing heavy clothing and 1 kg deducted for remaining clothing). Body mass index (BMI) was calculated as body weight (kg) by the square of the height (m^2^).

Venous blood samples were collected to measure blood glucose and serum cholesterol, high-density lipoprotein (HDL), low-density lipoprotein (LDL), uric acid and creatinine. Serum creatinine was measured by Jaffe’s kinetic method. In our hospital’s lab, the standard substance (SRM 909b) was used to calibrate the Jaffe’s kinetic method to IDMS method of the CKD-EPI eGFR. SUA was measured by oxidization method.

### Diagnosis

Hypertension was defined if the systolic blood pressure level ≥140 mmHg, or diastolic blood pressure ≥90 mmHg, self-reported history of hypertension, or use of antihypertensive drugs [13]. Diabetes was defined as fasting blood glucose level ≥7.0 mmol/L (126 mg/dl) or 2 h postprandial blood glucose ≥11.1 mmol/L (200 mg/dl) [14]. Body mass index (BMI) more than 30 (Kg/m^2^) was defined as obesity and BMI between 25 (Kg/m^2^) to 30 (Kg/m^2^) was overweight [15]. A blood hemoglobin level ≤130 g/L in men or women elder than 50 years was defined as anemia. Serum cholesterol level ≥221.36 mg/dl (5.72 mmol/L) and triglyceride ≥150 mg/dl (1.7 mmol/L) were defined as hypercholesterinemia and hypertriglyceridemia, respectively. The HDL level ≤40 mg/dl (1.04 mmol/L) in male or the HDL level ≤50 mg/dl (1.30 mmol/L) in female was defined as abnormality [16]. Hyperuricemia was defined as the SUA level ≥7.05 mg/dl (420 μmol/L) in male or ≥6.04 mg/dl (360 μmol/L) in female. According to the previous report [17], eGFR was calculated by CKD-EPI [18]. eGFR (ml/min/1.73 m^2^)=141 × min (Scr/κ, 1)^a^×max(Scr/κ, 1)^−1.209^×0.993^Age^×1.018 [if female] , where Scr is serum creatinine, κ is 0.7 for females and 0.9 for males, a is −0.329 for females and −0.411 for males, min indicates the minimum of Scr/κ or 1, and max indicates the maximum of Scr/κ or 1. According to the Kidney Disease Outcomes Quality Initiative (K/DOQI) classification system, eGFR <60 mL/min/1.73 m^2^ was defined as CKD [19].

### Statistical analysis

Data were presented as means ± standard deviation for continuous variables and as proportions for categorical variables. Differences between male and female subjects were examined using Pearson′s chi-square test for categorical variables and two-tailed paired Student's *t*-test for continuous data. SUA levels were examined as a continuous variable and as gender-specific quartiles. Quartiles 1–4 in male were 307 μmol/L, 308–359 μmol/L, 360–419 μmol/L and ≥420 μmol/L; quartiles 1–4 in females were ≤236 μmol/L, 237–276 μmol/L, 277–330 μmol/L and ≥331 μmol/L.

Multivariate binary logistic regression analysis was used to estimate odds ratios (ORs) and 95% confidence intervals (95%CIs) by comparing association between the SUA level and CKD. The models were adjusted for age, gender, BMI, hypertension, diabetes, triglyceride and HDL-C. *p* values <.05 were considered to be statistically significant. SPSS 11.5 software (SPSS Inc, USA) was used for data analysis.

## Results

Among the 4242 total subjects, the distributions of SUA levels showed gender difference. The prevalence of hyperuricemia was 22.2% in the total population, and the prevalence of hyperuricemia was significantly higher in male than in female (25.2% vs. 17%, *p*<.001). The prevalence of CKD was 27.8% in male and 20.2% in female (*p*<.001).

### Age and gender-specific prevalence of hyperuricemia

**Figure 1.**
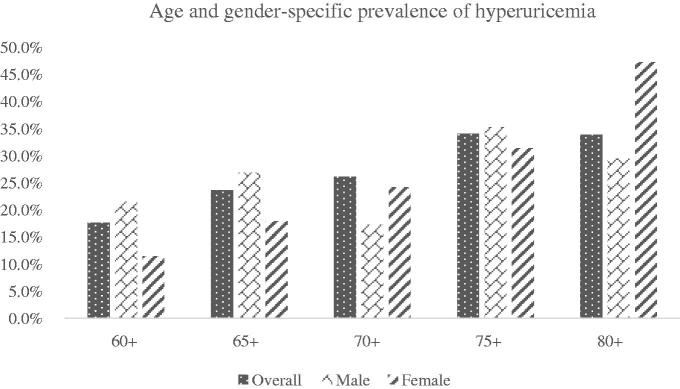
Age and gender-specific prevalence of hyperuricemia.

Figure 1 describes the prevalence of hyperuricemia by age and by gender. The prevalence of hyperuricemia showed an increasing trend with different age subgroups. Female had a higher prevalence of hyperuricemia in female subjects elder than 70 years. However, SUA level did not vary significantly in different age subgroups among male.

### Prevalence of CKD for each quartile of SUA

**Figure 2.**
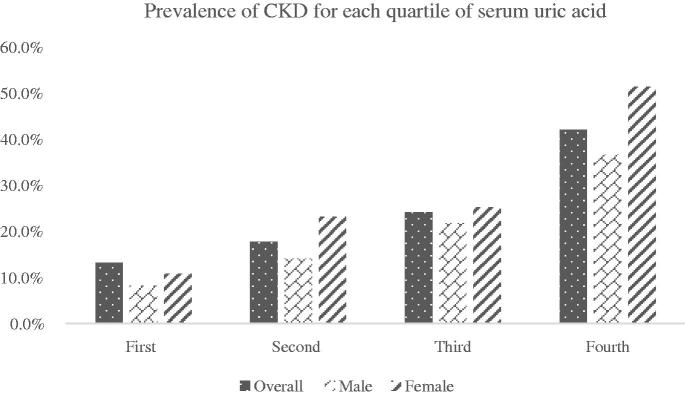
Prevalence of CKD for each quartile of serum uric acid.

Figure 2 describes the prevalence of CKD for each quartile of SUA. As SUA level increased, the percentage of CKD was increased from 13.2% in the first quartile to 42.1%% in the quartiles (*p* < .001). The prevalence of CKD showed an increasing trend with different gender group in quartiles. Female had a higher prevalence than male in the fourth quartile (51.4% vs. 36.6%, *p* < .001).

### Clinical characteristics by quartiles of uric acid levels in male and females

Subjects were categorized into four subgroups according to the quartiles of their SUA levels. Clinical characteristics of the male and female subjects were shown in Table 1 and Table 2. Compared to 1st quartile in males, the 4th quartile in male was elder and showed a higher level of BMI, SBP, glucose, triglycerides, BUN, creatinine as well as lower eGFR and HDL-C.

**Table 1.** Clinical characteristics by quartiles of uric acid levels in male.

	SUA quartiles				*p*
	Q1	Q2	Q3	Q4	
	≤307 ummol/L	308–359 ummol/L	360–419 ummol/L	≥420 ummol/L	
					
Uric acid	266.43 ± 33.53	334.27 ± 14.90	387.48 ± 17.64	482.42 ± 53.89	<.001
Age (years)	66.79 ± 6.11	66.88 ± 6.37	68.63 ± 6.25	68.46 ± 6.53	<.001
Body-mass index (kg/m^2^)	23.48 ± 2.92	24.19 ± 2.99	24.71 ± 3.13	25.26 ± 3.06	<.001
Systolic pressure (mmHg)	130.12 ± 18.31	131.14 ± 18.26	133.69 ± 18.60	134.66 ± 17.79	.005
Diastolic pressure (mmHg)	79.52 ± 9.86	80.03 ± 9.90	80.15 ± 9.55	80.70 ± 10.57	.726
Hemoglobin (g/L)	142.36 ± 12.82	143.51 ± 12.45	143.59 ± 12.58	143.62 ± 14.01	.001
Serum glucose (mmol/L)	6.09 ± 1.95	5.68 ± 1.30	5.71 ± 1.34	5.84 ± 1.53	.256
Cholesterol (mmol/L)	4.70 ± 0.80	4.71 ± 0.87	4.72 ± 0.87	4.78 ± 0.93	.066
Triglyceride (mmol/L)	1.22 ± 0.73	1.41 ± 1.18	1.54 ± 1.11	1.78 ± 1.09	<.001
HDL-C (mmol/L)	1.42 ± 0.37	1.37 ± 0.37	1.32 ± 0.34	1.27 ± 0.33	<.001
LDL-C (mmol/L)	2.73 ± 0.70	2.71 ± 0.74	2.70 ± 0.73	2.72 ± 0.79	<.01
Creatinine (umol/L)	76.59 ± 29.68	79.70 ± 13.11	84.16 ± 27.91	92.54 ± 35.86	<.001
eGFR(ml min^−1^ 1.73 m^−2^)	78.98 ± 11.31	75.28 ± 11.01	72.18 ± 11.21	67.22 ± 12.58	<.001

**Table 2.** Clinical characteristics by quartiles of uric acid levels in female.

	SUA quartiles				*p*
	Q1	Q2	Q3	Q4	
	≤236 ummol/L	237–276 ummol/L	277–330 ummol/L	≥331 ummol/L	
					
Uric acid	203.84 ± 26.61	256.35 ± 11.46	301.59 ± 15.59	394.66 ± 60.32	<.001
Age (years)	65.26 ± 5.29	66.29 ± 5.57	66.13 ± 5.71	68.42 ± 6.80	<.001
Body-mass index (kg/m^2^)	23.80 ± 7.53	24.36 ± 3.10	24.95 ± 3.40	25.97 ± 3.51	<.001
Systolic pressure (mmHg)	127.97 ± 17.95	129.15 ± 17.16	131.18 ± 19.05	132.14 ± 17.57	.007
Diastolic pressure (mmHg)	78.03 ± 9.62	77.56 ± 8.96	78.32 ± 9.38	77.89 ± 9.54	.925
Hemoglobin (g/L)	125.89 ± 10.32	127.22 ± 9.28	128.65 ± 9.23	126.25 ± 11.50	<.001
Serum glucose (mmol/L)	5.84 ± 1.66	5.75 ± 1.28	5.67 ± 1.16	5.82 ± 1.13	.837
Cholesterol (mmol/L)	5.10 ± 0.86	5.18 ± 0.87	5.13 ± 0.88	5.02 ± 0.89	.050
Triglyceride (mmol/L)	1.32 ± 0.78	1.47 ± 0.75	1.71 ± 1.25	1.87 ± 0.99	<.001
HDL-C (mmol/L)	1.53 ± 0.34	1.49 ± 0.33	1.42 ± 0.38	1.36 ± 0.29	<.001
LDL-C (mmol/L)	2.97 ± 0.72	3.03 ± 0.76	2.95 ± 0.74	2.82 ± 0.76	.933
Creatinine (umol/L)	57.52 ± 12.86	61.40 ± 9.45	62.34 ± 10.27	71.98 ± 21.06	<.001
Nitrogen (mmol/L)	5.57 ± 1.35	5.70 ± 1.33	5.63 ± 1.97	5.80 ± 1.54	<.001
eGFR(ml min^−1^ 1.73 m^−2^)	71.02 ± 9.81	66.61 ± 8.96	65.89 ± 8.78	59.11 ± 12.28	<.001

In female, compared to 1st quartile, 4th quartile was also elder and showed a higher level of BMI, SBP, triglycerides, LDL-C, BUN, creatinine as well as lower hemoglobin and HDL-C.

### Multivariate analysis of the association between serum and CKD in the entire population

The ORs of CKD by increasing SUA quartiles in the entire cohort were presented in Table 3. Univariate analysis showed (OR: 4.05, 95%CI: 3.26–5.04), after conducting the multivariable analysis adjusting for the potential confounding factors including gender, age, diabetes, HDL-C, hypertension, BMI, triglyceride, the Q4 quartiles of SUA are more associated with CKD comparing Q1 SUA (OR: 7.23, 95%CI: 5.48–9.53).

**Table 3.** Association between quartiles of uric acid and CKD in the entire population.

SUA quartiles	Unadjusted OR	95%CI	*p*	Adjusted OR	95%CI	*p* value
Q1 (≤272 ummol/L)	Reference			Reference		
Q2 (273 to ≤330 ummol/L)	1.41	1.11–1.78	*p*<.001	1.84	1.41–2.38	*p*<.001
Q3 (331 to ≤395 ummol/L)	1.88	1.49–2.37	*p*<.001	3.10	2.37–4.06	*p*<.001
Q4 (≥396 ummol/L )	4.05	3.26–5.04	*p*<.001	7.23	5.48–9.53	*p*<.001

Adjusted for gender, age, diabetes, HDL-C, hypertension, BMI, Triglyceride.

### Multivariate analysis of the association between SUA and CKD in male and female subjects

The ORs of CKD by increasing SUA quartiles in male and female are presented in Tables 4 and 5. After adjusting for the potential confounding factors including age, diabetes, HDL-C, hypertension, BMI, triglyceride, higher quartiles of SUA were significantly associated with CKD in both male and female subjects. Although comparing with Q1 SUA, the Q4 quartiles of SUA were associated with CKD in both male and female, the association between hyperuricemia and CKD was stronger in female than in male (OR: 6.05, 95%CI: 4.32–8.49; OR: 8.21, 95%CI 5.37–12.54).

**Table 4.** Association between quartiles of uric acid and CKD in male.

SUA quartiles	Unadjusted OR	95%CI	*p* value	Adjusted OR	95%CI	*p* value
Q1 (≤307 ummol/L)	Reference			Reference		
Q2 (308 to ≤358 ummol/L)	1.82	1.29–2.59	*p*<.001	1.80	1.25–2.60	*p*<.001
Q3 (359 to ≤419 ummol/L)	3.10	2.23–4.32	*p*<.001	2.93	2.07–4.16	*p*<.001
Q4 (≥420 ummol/L)	6.42	4.68–8.79	*p*<.001	6.05	4.32–8.49	*p*<.001

Adjusted for age, diabetes, HDL-C, hypertension, BMI, Triglyceride.

**Table 5.** Association between quartiles of uric acid and CKD in female.

SUA quartiles	Unadjusted OR	95%CI	*p* value	Adjusted OR	95%CI	*p* value
Q1 (≤236 ummol/L)	Reference			Reference		
Q2 (237 to ≤276 ummol/L)	2.49	1.67–3.72	*p*<.001	2.46	1.60–3.78	*p*<.001
Q3 (277 to ≤330 ummol/L)	2.79	1.88–4.15	*p*<.001	2.87	1.87–4.39	*p*<.001
Q4 (≥331 ummol/L )	8.72	5.96–12.76	*p*<.001	8.21	5.37–12.54	*p*<.001

Adjusted for age, diabetes, HDL-C, hypertension, BMI, Triglyceride.

## Discussion

In the present study, we reported that the prevalence of hyperuricemia was 22.2% in the entire elderly population. The prevalence of hyperuricemia was significantly higher in elderly male than in elderly female. The prevalence of hyperuricemia increased with the age, especially in elderly population female. The higher SUA level was associated with increased risk of CKD.

Hyperuricemia can result from either increased uric acid synthesis or decreased uric acid excretion, or from a combination of both [20]. Kamei et al [21] found that SUA levels in female were lower than in male. Age-related increase in SUA levels was found among women, in contrast, SUA level did not vary significantly among man [22]. A survey based on individuals elder than 65 years suggested the prevalence of hyperuricemia increased of only 6% in men from 65 to over 90 years, but doubled in women (from 15.3% in those having 65 years to 34.4% in the 90s) [23]. In the present study, we found the prevalence of hyperuricemia was 22.2% in the elder population, with 25.2% in male and 17.0% in female. After we calculated the age-specific prevalence, we found that male had a higher prevalence of hyperuricemia in subjects younger than 70 years, while in the population elder than 70 years, the female subjects had the similar prevalence of hyperuricemia as male. We also found that SUA level did not vary significantly in different age subgroups among elderly male. Potential explanations include (1) alcohol consumption, which is higher in male; (2) the influence of estrogens. Estrogens promote renal uric acid excretion and decrease the level of SUA by suppressing the protein levels of the urate reabsorptive urate transporter 1 (URAT1) and glucose transporter 9 (GLUT9) in the proximal tubule, and that of urate efflux transporter ATP-binding cassette sub-family G member 2 (ABCG2) [24]. Postmenopausal hormone use is associated with lower uric acid levels among postmenopausal women [25].

Weiner et al [11] analyzed data from the two cohort studies, the Atherosclerosis risks in Communities (ARIC) and the Cardiovascular Health Study (CHS). They found the elevated SUA level was a modest independent risk factor for incident kidney disease. This analysis involved 13,338 subjects following for a mean period of 8.5 years. Similar findings were reported in other previous studies [26]. A systematic review and meta-analysis study, containing 190,718 participants, showed that a significant positive association between elevated SUA levels and the new-onset CKD. Hyperuricemia was found to be an independent predictor for the new onset CKD [27]. Prevalence of CKD also increases in age [28]. Hyperuricemia may be one of the strongest risk factors related to CKD in elderly population [29]. The elderly population has a higher level of SUA and a high risk of developing CKD [12]. In previous studies, the association between the level of uric acid and CKD in different gender was evaluated. Mixed results were obtained in elderly population [5,9–11]. In the previous cross-sectional study, Li et al [9] reported that the association between hyperuricemia and CKD was stronger in elderly male (OR (95% CI): 2.04 (1.56–2.67), *p* < .001) than in elderly females (1.45 (1.17–1.80), *p* = .001). Similar result was obtained in an investigation including 24.886 elderly residents in a community [10]. A retrospective study of IgAN patients showed no significant differences observed in renal survival between male and female by SUA level-matching [30]. Another single-center retrospective cohort study showed that an elevated uric acid level was an independent risk factor for ESKD in female IgA patients but not male patients [5]. Weiner also found that the baseline SUA was associated with increased risk of CKD in female and a trend in male [11]. Recently in a study [31], a gender-specific association between the elevated uric acid level and CKD was analyzed in 3702 African Americans. In this study, the average age of subjects was 55.25 ± 12.40 years. The result indicated that both new-onset CKD (OR: 1.96; 95%CI: 1.11–3.46) and the rapid progression of CKD (OR: 2.06; 95%CI: 1.25–3.37) were more likely to occur among the female participants with the elevated uric acid level. However, the similar association of the elevated uric acid level with CKD was not found among the male participants with the elevated uric acid [31]. The interesting of our present study was that, despite the presence of lower concentration of SUA in female, the association between hyperuricemia and CKD in female was significantly stronger than that in males. The adjusted ORs of hyperuricemia for CKD were 6.16 (4.39–8.64) for male and 8.32 (5.48–12.63) for female, respectively. The risk of renal arteriolar hyalinosis and developing renal insufficiency above the cutoff value of UA might differ between the sexes, which were higher for male than for female [32]. Hyperuricemia was associated with an increased risk of incident hypertension pronouncing in younger female [33]. The mechanisms that cause hyperuricemia to be less related to CKD in elderly male than female remain uncertain and providing a potential explanation for it would be rather speculative.

There were some limitations to the present study. First, although 2012 KDOQI guideline recommended CKD-EPI equation for calculating eGFR in adults [34], the CKD-EPI equations were developed in a North American and European study population. Whether the CKD-EPI was superior to the other eGFR equations need to be identified in Chinese elderly community-dwelling. Second, the cross-sectional study could not identify potential causality between hyperuricemia and CKD. The further cohort study is needed to verify the association. Third, the aged population has a higher prevalence of hypertension and cardiovascular diseases which might have a synergic impact on the kidney. Fourth, we could not collect information on medication use, dietary intake and alcohol drinking. Above factors might impact on the results.

In conclusion, we evaluated the association between SUA and CKD in a Chinese dwelling community elderly population. We found that gender-related differences in the association between high SUA and an increased risk of CKD in the population after 60 years of age. The association of SUA and CKD was independent of other potential confounding factors including age, glucose, hypertension, HDL, TG and BMI.
